# Herbicide Resistance in *Phalaris* Species: A Review

**DOI:** 10.3390/plants10112248

**Published:** 2021-10-21

**Authors:** Javid Gherekhloo, Saeid Hassanpour-bourkheili, Parvin Hejazirad, Sajedeh Golmohammadzadeh, Jose G. Vazquez-Garcia, Rafael De Prado

**Affiliations:** 1 Department of Agronomy, Gorgan University of Agricultural Sciences and Natural Resources, Gorgan 49189-43464, Iran; s.hassanpour.b@gmail.com (S.H.-b.); parvinhejazirad2012@gmail.com (P.H.); sa_gmz@yahoo.com (S.G.); 2 Department of Agricultural Chemistry, Edaphology and Microbiology, University of Cordoba, 14071 Cordoba, Spain; z82vagaj@uco.es

**Keywords:** fitness cost, resistance management, resistance mechanism, weed, world distribution

## Abstract

Weeds, such as *Phalaris* spp., can drastically reduce the yield of crops, and the evolution of resistance to herbicides has further exacerbated this issue. Thus far, 23 cases of herbicide resistance in 11 countries have been reported in *Phalaris* spp., including *Phalaris minor* Retz., *Phalaris paradoxa* L., and *Phalaris brachystachys* L., for photosystem II (PS-II), acetyl-CoA carboxylase (ACCase), and acetolactate synthase (ALS)-inhibiting herbicides. This paper will first review the cases of herbicide resistance reported in *P. minor*, *P. paradoxa*, and *P. brachystachys*. Then, the mechanisms of resistance in *Phalaris* spp. are discussed in detail. Finally, the fitness cost of herbicide resistance and the literature on the management of herbicide-resistant weeds from these species are reviewed.

## 1. Introduction

*Phalaris* species grow in various environments, including wild communities to disturbed areas in arable lands, sandy soils, and waste beds [1]. Most *Phalaris* species are weeds that infest winter crops and prefer heavy clay soils [2]. Presently, 22 species of *Phalaris* have been recognized [3], out of which, *Phalaris minor* Retz., *Phalaris paradoxa* L.), and *Phalaris brachystachys* L. are among the most important weeds in agricultural systems. These species are common weeds in wheat fields across the world [4].

Weed control is among the key components of crop systems, which, if not performed properly, will result in significant performance and financial loss for producers [3]. The application of herbicides is currently a crucially important management strategy in crops. Presently, approx. 150 chemical compounds are used to control weeds, representing 25 different sites of action in total [5]. Since the 1970s, many cases of resistance development have been documented due to repeated herbicide applications for weed control. The evolution of herbicide resistance in weeds depends on various factors, such as weed biology, ecology, and genetics, as well as herbicide application [6]. Currently, 505 biotypes in 263 species, comprising 266 dicotyledons and 239 monocotyledons, have developed resistance to herbicides in 93 crops worldwide [7].

Photosystem II (PS-II), acetyl-CoA carboxylase (ACCase), and acetolactate synthase (ALS)-inhibiting herbicides are commonly used to control grass weeds in wheat [8,9]. However, the consecutive use of these herbicides has led to the evolution of resistant biotypes of these species (discussed in detail below). *Phalaris* species are considered to have a medium inherent risk of evolving resistance [10]. To date, there have been at least 23 reports of herbicide resistance in this species, with multiple and cross-resistance reported [7]. Herbicide resistance in *Phalaris* spp. has been reported for three modes of action: namely, PS-II, ACCase, and ALS inhibitors, and has been described in *P. minor*, *P. paradoxa*, and *P. brachystachys* [7]; the development of herbicide-resistant *Phalaris* species in fields may be a serious threat to crop yields and sustainable wheat production and may also threaten the biodiversity of accompanying weed communities [3,11].

These three species are frequently found in similar types of agroecosystems, mainly winter cereals, where they cause the greatest yield losses. Yield losses in wheat due to *P. minor* alone may be up to 50%, and heavy infestations may lead to total crop failure [8,12]. Wheat crop biomass was significantly reduced (14.8%) with an infestation of 200 *P. minor* plants/m^2^. *P. paradoxa* grows taller than some cereal crops (wheat and barley), and 100 plants of *P. paradoxa* were sufficient to significantly reduce the wheat yield about 17.4% [13,14]. The economic threshold levels of *P. minor* and *P. paradoxa* were reported to be 3.1 and 2.6 plants/m2, respectively, for manual weeding in China [15]. *P. brachystachys* reduced the wheat crop yield by 36 percent when 152 plants/m^2^ were planted [16]. Presently, there are no exhaustive reviews available on resistance cases, the mechanisms of resistance, relative fitness, and management of resistance in *Phalaris* spp. The aims of this paper are (i) an overview of the status of herbicide resistance reported in *P. minor*, *P. paradoxa*, and *P. brachystachys* and their mechanisms of resistance and (ii) review the fitness cost of herbicide resistance and the literature on the management of herbicide-resistant weeds from these species.

## 2. Biology and Distribution of *Phalaris* spp.

The genus *Phalaris* is grown mostly in the Mediterranean climate. *P. minor* (2n = 28)*, P. brachystachys* (2n = 12), and *P. paradoxa* (2n = 14) are self-pollinated annual grass weeds. *P. minor* has been reported in more than 60 countries and is found in all the continents except the polar regions [17]. This weed infests wheat fields in India and Pakistan, the Mediterranean countries, the Arabian Peninsula and the Middle East, Central America, Australia, and South Africa [18]. *P. minor* is a competitive weed species that infests various crops. The problem is very acute in vast areas of South Asia, where rice–wheat cropping systems are common. *P. brachystachys* is native to the Madeira Islands, Canary Islands, and temperate Asia; it is naturalized in Southern Europe, North Africa, and North America [18]. *P. paradoxa* is native to Southwest Europe and the Mediterranean (including Northern Africa and Western Asia) but has spread to other regions, including the United States, Australia, and South America. It is a serious weed of wheat in Australia, with its success attributed to a high seed production, innate dormancy, and periodicity of emergence [19]. *P. paradoxa* is considered to be the third-most difficult grass weed of wheat and winter pulse crop production systems in subtropical Australia [20].

These annual grass species reproduce through seed production and shed seeds before or during crop harvesting, thus increasing the size of the soil weed seed bank. Seed widths and lengths and 1000-seed weights were different across these three species. *P. brachystachys* seed widths and lengths and 1000-seed weights were greater than *P. minor* and *P. paradoxa* [21]. These weeds generally produced 10–50 spikes per plant and generated a large number of seeds in wheat fields [22]. Each plant of *P. minor* can produce around 5000 or even more seeds, depending on the number of spikelets produced [23]. The *P. minor* seeds require 4 to 5 months of after-ripening to attain maximal germination after dispersal [21]. The seed germination of fresh seeds of *P. brachystachys* was less than *P. minor* and *P. paradoxa*; the germination of *P. brachystachys* increased with enhancement of the GA_3_ concentration (400 ppm) [21]. The seed germination increased by about 6–8 months after-ripening. The seed germination of *P. paradoxa* was nearly 95% within 2 months after being harvested [24]. A large proportion of these species seeds germinate between mid-November and mid-December. The spread and establishment of these three species can occur in soils with pH ranges from 4 to 8 [21]. The germination in *Phalaris* spp. was much lower in the dark (on average, 13%) than in the light (on average, 76%) [19,25]. It was also reported that their seed germination adapted to low temperatures. There are significant differences in leaf characteristics and growth habits between wheat and *Phalaris* species [25]. The tillering and branching occur in *Phalaris* species, while it does not occur in wheat or barley. Weed risk assessment studies have categorized *P. paradoxa* under the “high risk and invasive” category for the United States [26] and India [27]. The result of the weed risk assessment for *P. brachystachys* in the United States is high risk [28].

## 3. Mechanisms of Herbicide Resistance

An essential aspect of predicting the evolutionary course of herbicide resistance traits is understanding the mechanism(s) of herbicide resistance. Knowledge of the resistance mechanisms is essential for developing effective weed management strategies to control and delay the onset of herbicide resistance [29]. The mechanisms of herbicide resistance in weeds can be broadly classified into target site resistance (TSR) and non-target site resistance (NTSR) [30].

TSR mechanisms change the target enzyme’s amino acid sequence and/or expression level, decreasing the herbicide’s ability to inhibit it. Thus, a higher herbicide concentration may be required to achieve sufficient inhibition in TSR. A single amino acid alteration in the gene encoding an herbicide-binding protein can disrupt the herbicide’s ability to bind to the protein without affecting the enzyme’s function and may impose a fitness cost [30]. Whether a specific target site mutation that confers resistance to a particular herbicide also confers resistance to other chemical families within the same site of action group depends on how the specific herbicides interact with the target protein [29]. Another type of TSR involves the expression of the gene at the target site that produces more enzymes than can be substantially inhibited by the typical herbicide application rates. An increased gene expression may be due to regulatory changes that increase the transcription and/or an increase the genomic copy number of the gene at the target site, which also leads to increased transcription. Most, but not all, cases of herbicide resistance to ALS inhibitors, ACCase, triazine, dinitroaniline, and other herbicides are caused by changes in the herbicide’s site of action [30].

NTSR includes any mechanism that reduces the concentration of an active herbicide that remains available to interact with the target site protein, as well as mechanisms that allow the plant to cope with inhibition of the target site [31]. These mechanisms include increased herbicide sequestration, reduced herbicide uptake and translocation, and increased degradation or metabolism of the herbicide to compounds with less toxicity. Four enzyme families: cytochrome P450 monooxygenases (CYP450; EC 1.6.2.4), glutathione-S-transferases (GSTs; EC 2.5.1.18), glycosyltransferases (GTs; EC 2.4), and ABC transporters are involved in herbicide resistance in NTSR [32,33]. The CYP450, GST, and GT enzyme families are involved in the biochemical modification of herbicides through the metabolism, while ABC transporters mediate herbicide resistance by compartmentalizing herbicides and their metabolites [34].

## 4. Herbicide Resistance in *Phalaris* spp. and Their Mechanisms

Populations of *Phalaris* spp. have evolved a variety of resistance mechanisms, including mutation and enhanced herbicide metabolism. Table 1 summarizes the current worldwide occurrence of *Phalaris* spp. with resistance to different herbicide groups.

### 4.1. Resistance to PSII Inhibitors

Several chemical herbicide classes (e.g., triazines, triazinones, ureas, uracil, phenylcarbamates, and amides) inhibit PSII, which competes with plastoquinone (PQ) for the PQ-binding site on the D1 protein encoded by the psbA gene, thereby inhibiting PSII electron transport. Isoproturon and triazine herbicides are known inhibitors of PSII and bind to the D1 protein of the PSII reaction center [37]. This blocks the electron transfer from plastoquinone QA in D2 to plastoquinone QB in D1, preventing CO_2_ fixation and the production of ATP and NADPH [53].

Blocking the electron transport leads to reactive oxygen species (ROS) production, which destroys the cell integrity. The resistance to PSII-inhibiting herbicides is primarily caused by two mechanisms: TSR and NTSR. The TSR mechanism is caused by amino acid substitutions in the PSII complex’s D1 protein, which is encoded by the chloroplast psbA gene. Of the total 345 amino acids in the D1 protein, approximately 60 are part of the herbicide and QB-binding site. Several amino acid substitutions conferring resistance to herbicide PSII inhibitors have been identified in or near the QB-binding site [37]. To date, eight psbA gene mutations, including Ser-264-Gly, Ser-264-Thr, Val-219-Ile, Asn-266-Thr, Ala-251-Val, Phe-255-Ile, Leu-218-Val, and Phe-274-Val, have been reported in field-developed PSII inhibitor-resistant weed species [53]. However, there are some cases where the resistance is NTSR. In these cases, resistance is due to increased metabolism [37,38,54,55].

The first confirmed herbicide-resistant *Phalaris* spp. was *P. minor*, which developed a resistance to PSII inhibitors (isoproturon) in wheat fields in India in 1991 [7]. Phenylurea herbicides (metoxuron, methabenzthiazuron, and isoproturon) were recommended to control *P. minor* in wheat in the rice–wheat cropping system in India [56]. The consecutive application of isoproturon for 10–15 years in a monoculture cultivation of rice–wheat led to the development of herbicide-resistant biotypes of *P. minor* in India [37,38,57,58,59]. The response of resistant biotypes of *P. minor* to other phenylurea herbicides, such as methabenzthiazuron and metoxuron, was similar to that of isoproturon, confirming the resistance to other phenylurea herbicides [60]. After the development of isoproturon resistance, four alternative herbicides such as sulfosulfuron, clodinafop-propargyl, fenoxaprop-P-ethyl, and tralkoxydim were recommended to control the *P. minor* isoproturon-resistant population. A multiple resistance in the isoproturon-resistant *P. minor* populations to diclofop-methyl, pinoxaden, and sulfosulfuron was reported. Populations that were resistant to multiple herbicides showed a low level of resistance to sulfosulfuron, a moderate level of resistance to pinoxaden, and a high level of resistance to clodinafop-propargyl and fenoxaprop-P-ethyl [42]. Altogether, the *P. minor* populations were resistant to six herbicide mode of action groups (phenylurea, sulfonylurea, aryloxyphenoxypropionic, cyclohexenoxime, phenylpyrazole, and triazolopyrimidine sulfonamide) [61]. In *P. minor*, the GR_50_ levels of sulfosulfuron, fenoxaprop-P-ethyl, and clodinafop-propargyl were increased 10-, 8-, and 4-fold compared to the susceptible population [62]. Atrazine-resistant biotypes of *P. paradoxa* were reported in Israel [63,64].

Several studies have been conducted on the mechanism of isoproturon resistance in *P. minor* populations [37,38,54,55]. Modification of the amino acid residues in the QB-binding site on the D1 protein conferred TSR to isoproturon in *P. minor* [65]. A single Ser-264-Gly mutation in the psbA gene was found in triazine-resistant *P. paradoxa* [63]. Apart from TSR, studies on herbicide metabolism and CYP450 inhibitors in resistant biotypes found that the activity of CYP450 increased in isoproturon-resistant biotypes of *P. minor* [66]. The degradation of ^14^C-isoproturon was faster in the resistant biotype of *P. minor* than the susceptible biotype, but the uptake and translocation of isoproturon did not vary between the resistant and susceptible biotypes [54,55]. The isoproturon treatments when applied with CYP450 inhibitor PBO significantly reduced the dry weight of the resistant biotypes of *P. minor*, and the mechanism of resistance may be due to enhanced metabolism [38]. The absence of mutations in the herbicide-binding region of the psbA gene of isoproturon *P. minor*-resistant biotypes suggests that the target site resistance mechanism is not responsible for the resistance. Thus, the authors assumed this resistance must be caused by a NTSR mechanism [37].

### 4.2. Resistance to ACCase Inhibitors

ACCase (EC 6.4.1.2) is a key enzyme for fatty acid biosynthetic pathways. Two forms of ACCase occur in plants: prokaryotic and eukaryotic forms. The prokaryotic from is insensitive to herbicides and is found only in the plastids of dicotyledonous plants [31]. The eukaryotic form of ACCase found in the cytoplasm and the plastids of grasses is inhibited by the chemical families of the herbicides aryloxyphenoxypropionate (APP), cyclohexanedione (CHD), and phenylpyrazoline (PPZ) [67]. The ACCase inhibitor herbicides were first introduced in the late 1970s. ACCase inhibitors provide excellent weed control in both cereals and dicotyledonous crops. Resistance to ACCase inhibitor herbicides in *Phalaris* spp. is widespread. It was first identified in *P. minor* in India [7], which was followed by resistance observations in other countries. The resistance of *P. minor* to fenoxaprop-P-ethyl was reported from a wheat field in Israel in 1993 [44]. This biotype required 20 times the amount of fenoxaprop-P-ethyl to achieve the same level of control as the susceptible biotype. A low level of resistance was also observed with ACCase inhibitor herbicides, such as diclofop-methyl, clodinafop-propargyl, sethoxydim, and tralkoxydim [52]. A *P. minor* population from South Africa was shown to be resistant to clodinafop-propargyl and diclofop-methyl [49]. Resistance to ACCase-inhibiting herbicides in *Phalaris* spp. has been previously reported in *P. paradoxa* [46,50,51], *P.* minor [35,40,46,47,51,52,68,69,70,71,72,73,74], and *P. brachystachys* [4].

TSR in ACCase-inhibiting herbicides was essentially caused by a single amino acid substitution at any of these seven positions (1781, 1999, 2027, 2041, 2078, 2088, and 2096). These substitutions may occur in one position or more than one position in the ACCase gene, which may confer different resistance patterns among the ACCase inhibitors [67]. Amino acid substitutions leading to resistance to ACCase-inhibiting herbicides in different populations of *P. minor* have also been identified, including amino acids at 1781 [35,40], 2027 [23,40,41,75], 2041 [23,41], and 2078 [45,51]. In these cases, a substitution rendered ACCase insensitive to graminicides in the resistant *P. minor* population and conferred different resistance patterns. The Ile-1781-Leu and Asp-2078-Gly substitutions conferred a cross-resistance to herbicides APP, CHD, and PPZ in *P. minor* from Mexico [35]; Trp-2027-Cys and Asp-2078-Gly substitutions have also been reported in Iranian *P. minor* populations, conferring resistance to ACCase inhibitors and conferring resistance to APPs (fenoxaprop-P-ethyl, clodinafop-propargyl, and diclofop- methyl) [45]. The Trp-2027-Cys and Ile-2041-Asn mutations in *P. minor* from India conferred resistance to clodinafop-propargyl. The Trp-2027-Cys mutation also conferred resistance to pinoxaden, while the Ile-2041-Asn mutation conferred a moderate resistance to pinoxaden [45].

In the literature, there are only three reports of ACCase TSR in *P. paradoxa* in Israel, Italy, and Mexico. The two mutations, Asp-2078-Gly and Ile-2041-Asn, reported in *P. paradoxa* and field trials showed that one population was highly resistant to all the studied ACCase-inhibiting herbicides and also showed a cross-resistance to herbicides APP, CHD, and PPZ [49]. Two substitutions, Ile-1781-Val and Asp-2078-Gly, were found in the different resistant biotypes of *P. paradoxa* from Italy, which were possibly responsible for the resistance to herbicides APP and CHD, as well as pinoxaden (PPZ) resistance [50]. A Gly-2096-Ser substitution was found in the resistant *P. paradoxa* biotype from Mexico [51]. The substitution of Ile-1781-Thr in the resistant biotypes of *P. brachystachys* conferred a cross-resistance to APP and CHD and moderate resistance to pinoxaden. NTSR was present in biotypes already containing TSR alleles. CYP450-mediated enhanced metabolism plays a role in diclofop-methyl resistance in the resistant biotype of *P. brachystachys*, but the uptake and translocation did not vary between the resistant and susceptible biotypes [76]. A metabolic resistance in *Phalaris* spp. was convincingly confirmed for the first time in *P. brachystachys* with radiolabeled ^14^C herbicides [71], whereas no differences in the metabolisms of *P. minor* and *P. paradoxa* were reported [35,51].

### 4.3. Resistance to ALS Inhibitors

ALS is the first enzyme in the biosynthetic pathway to produce the branched-chain amino acids isoleucine, leucine, and valine. Five different chemical groups are known as ALS inhibitor herbicides: imidazolinone, sulfonylurea, pyrimidinyl benzoates, sulfonanilides, triazolinones, and triazolopyrimidine. These herbicides are used in almost all cropping systems, with wide variations in their selectivity, control spectrum, and residual activity. *P. minor* biotypes from India were resistant to iodosulfuron-methyl-sodium and mesosulfuron-methyl and may also be cross-resistant to other ALS herbicides [43]. A low level of resistance to clodinafop-propargyl, sulfosulfuron, fenoxaprop-P-ethyl, and tralkoxydim have been reported in *P. minor* [77]. The resistance of *P. minor* to clodinafop-propargyl and sulfosulfuron was also reported [78]. *P. brachystachys* biotypes from Turkey were resistant to clodinafop- propargyl and pyroxsulam [7]. In *P. minor* populations from South Africa, a resistance to multiple ACCase and ALS inhibitors has also been reported [36]. For these resistant biotypes, the mechanism of resistance has yet to be identified. Theoretically, the risk of cross- and multiple-resistances as a result of an herbicide metabolism may be higher than that of TSR [34]. Most cases of resistance in *Phalaris* spp. with an unknown mechanism of resistance reviewed in the present paper are cross- and multiple-resistant, and it may be hypothesized that herbicide metabolism may also be responsible for resistance in these cases, especially in cases that show a moderate resistance [34]. However, further experiments must be conducted to work out the mechanism(s) of resistance in the mentioned cases.

To summarize, cases of herbicide-resistant *P. minor*, *P. paradoxa*, and *P. brachystachys* have been reported in 11 countries. Of 23 cases, one case was resistant only to ALS inhibitors, and two cases developed a resistance only to PSII inhibitors, whereas the majority of the cases (15 cases) were ACCase-resistant. Furthermore, one case had multiple resistances to the ACCase and PSII inhibitors, and three were resistant to the ALS and ACCase inhibitors. However, there are no reports of multiple resistances to the PSII and ALS inhibitors. Additionally, there was one report on the resistance to multiple ACCase, ALS, and PSII inhibitors. However, the response of these resistant biotypes to other herbicide families commonly use in wheat should be investigated to further illustrate the resistance pattern, especially in cases with no reports on the mechanism of resistance.

## 5. Fitness Cost of Herbicide Resistance in *Phalaris* spp.

The evolution of resistance to herbicides may impose a fitness cost on weeds [79]. This fitness cost may be described as the reduction in the relative fitness of a species as a result of pleiotropic or direct effects, which may be imposed by resistance alleles [80] and may be considered as the final outcome of the changes in the genetic, biochemistry, and physiology of a weed due to resistance-conferring mutation(s) [81]. It may also be defined as the average success of a phenotype in the production of offspring in comparison to another phenotype [82]. Resistant plants show a greater fitness in comparison to susceptible ones under the selection pressure imposed by the herbicide to which the plant has developed resistance. However, once the herbicide selection pressure is removed, the resistant plants may exhibit a fitness cost [31,83,84].

The fitness cost of herbicide resistance may occur for the following reasons: (1) mutations in the gene encoding the herbicide target enzyme may disrupt the plant function and metabolism [81], (2) the resources required for growth and propagation may be rerouted to defense due to the evolution of resistance [85], and (3) resistant alleles may result in pleiotropic effects, which might adjust ecological relationships. For instance, the plant may become less attractive for pollinators due to higher concentrations of some secondary metabolites [86,87,88,89]. The fitness cost imposed by herbicide resistance may be quantified by measuring various characteristics of the species, including germination [83], phenology, vegetative characteristics, fecundity, and yield [90]. Conversely, herbicide resistance may impose no fitness cost on the species [91,92]. Furthermore, the mutation responsible for resistance may even lead to positive [93] effects on the growth and reproduction of the species. This outcome depends on the mutation and the species [82].

Only a limited number of studies are available regarding the fitness cost of herbicide resistance in *Phalaris* spp. The fitness cost of triazine resistance in susceptible and resistant *P. paradoxa* biotypes collected from Israel with Ser-264-Gly substitution were investigated [48]. They reported that the quantum yield of the resistant biotype was 30% lower than that of the susceptible one. Furthermore, the CO_2_ uptake and dry weight of the resistant and susceptible biotypes were similar, and the triazine-resistant *P. paradoxa* biotype had a higher germination and seedling vigor than the susceptible biotype [48]. The Ser-264-Gly mutation has been identified in *Amaranthus powellii* S.Wats. [94], *Echinochloa crus-galli* (L.) Beauv [95], *Vulpia bromoides* (L.) Gray [96], and *Raphanus raphanistrum* L. [97]. *A. powellii* with impaired photosynthesis due to the psbA mutation (Ser-264-Gly) has a higher leaf N concentration [94]. The atrazine-resistant accession of *Arabidopsis* showed a reduction in the photosynthetic yield and reduced growth that has been attributed to the reduced PSII electron transfer efficiency caused by the psbA mutant allele (Ser-246-Gly), whereas resistant accession had a higher electron transport compared with the sensitive accession at a lower temperature [98].

The response of the isoproturon-resistant biotype was similar to the susceptible one regarding the tiller number in the absence of herbicide selection pressure. However, the resistant biotype had a greater plant height and dry weight compared to the susceptible biotype. The mechanism of resistance was not tested in the paper, although the researchers attributed this resistance to herbicide degradation by CYP450 enzymes [8].

Investigating the fitness cost of the resistance to ACCase inhibitors in *P. minor*, biotypes collected within wheat fields in Mexico showed that the phenological stages in biotypes with Ile-2041-Asn and Ile-1781-Leu mutations were accelerated. However, these biotypes exhibited a reduction in total dry matter accumulation compared with the biotypes with Asp-2078-Gly and Trp-2027-Cys mutations. The latter two also had higher absolute and relative growth rates. The net assimilation rate of the biotypes was similar. Additionally, Asp-2078-Gly and Trp-2027-Cys biotypes had a greater leaf area duration due to a higher leaf area, leaf number, and biomass accumulation in leaves [23]. The seed embryo size of the biotypes with the Ile-2041-Asn and Ile-1781-Leu mutations was not statistically different from that of the susceptible biotype, whereas the embryo size was much smaller in the Asp-2078-Gly and Trp-2027-Cys biotypes. Furthermore, the germination rate and seed longevity of all the resistant biotypes were significantly lower than those of the susceptible biotype. Ile-2041-Asn had the highest germination rate and seed longevity compared to the susceptible biotype, followed by the Asp-2078-Gly, Ile-1781-Leu, and Trp-2027-Cys biotypes [99]. Due to the increased germination rate, the susceptible biotype had a greater canopy cover, competition intensity index, and relative productivity compared with the resistant biotypes. However, when the germination of resistant and susceptible biotypes was synchronized, the performance of the biotypes was similar [100].

*P. brachystachys* biotypes with the Ile-1781-Thr mutation were collected within wheat fields in Iran, and the CYP450-mediated NTSR mechanisms were studied [101]. The results showed that the ACCase-resistant biotypes had a higher germination percentage and rate compared to the susceptible biotype. However, no differences were observed among the resistant and susceptible biotypes regarding the cardinal temperatures for germination. Biotypes with both TSR and NTSR mechanisms had lower base water potentials (ψb_50_) (i.e., higher drought tolerance) compared to the susceptible biotype and biotypes with TSR as the sole resistance mechanism. Resistant biotypes had higher germination in response to NaCl concentrations compared to the susceptible biotype, whereas the germination of resistant and susceptible biotypes was similar under different pH conditions. The results of the seed burial at different soil depths showed that the emergence percentage of the resistant biotypes was greater than that of the susceptible biotype. Furthermore, the plant height, area, leaf number, dry weight, leaf area index, leaf area ratio, net assimilation rate, crop growth rate, spikelet per plant, spikelet length, and grains per m^2^ of the resistant biotypes were significantly higher than those of the susceptible biotypes when grown as monocultures. However, the relative growth rate, 1000-grain weight, and grain area were similar among the resistant and susceptible biotypes [101].

Interestingly, the evolution of herbicide resistance in most *Phalaris* spp. populations has resulted in a fitness benefit rather than a fitness cost. However, further studies are required to understand the reason behind this fitness benefit and its implications for the management of this species.

## 6. Management of Herbicide Resistance in *Phalaris* spp.

The evolution of herbicide resistance in weeds severely threatens sustainable agriculture, as it may result in reduced crop yield and quality and increased production costs [102]. Therefore, devising plans to address this issue is highly crucial [103], and numerous research and review papers are available on the management of herbicide-resistant weeds [104,105,106,107,108,109].

In order to minimize the contamination in the environment, it is necessary to reduce the herbicide inputs. One of the best methods to achieve this task is to integrate chemical and nonchemical control methods. Integrated weed management (IWM) approaches using good agronomic practices and competitive crops and varieties is a suitable control measure against resistant weeds [5]. The management of *P. minor* resistance to isoproturon may be carried out by crop rotation. Isoproturon-resistant *P. minor* was found in two-thirds of the fields under rice–wheat rotation [8], and including rice–berseem, sunflower, vegetable, cotton, and pigeon pea in the rotation drastically reduced the frequency of this herbicide-resistant weed. The introduction of sugarcane in a crop rotation can be vastly helpful in this regard due to its smothering effects on *P. minor* [109]. Furthermore, it allows farmers to use herbicides, such as simazine and atrazine, to manage isoproturon-resistant *P. minor*.

Planting wheat early, adopting no tillage strategies, placing rice straw mulch between the rows, and planting wheat varieties with early canopy closure and a greater accumulation of dry matter and crop rotation are among the nonchemical management methods to control *P. minor* populations resistant to isoproturon in wheat fields in India. An increased seeding rate and closer row spacing also controlled isoproturon-resistant *P. minor* in several wheat fields [74]. The management of isoproturon-resistant *P. minor* was also discussed in several other studies [105,106,107].

Introducing allelopathic crops in rotation as cover crops or water extract application offers ecofriendly and cost-effective weed control. The allelopathic potential of crops could be exploited to lower the number of weeds. Aqueous extracts; residues; and mulches of sorghum, rice, sunflower, and maize reduced the fenoxaprop-P-ethyl-resistant *P. minor* biomass in wheat by 48–100%, 48–100%, and 20–54%, respectively, and provided an acceptable level of weed control. These allelopathic extracts resulted in hormesis in the growth of the weed when applied at low concentrations [110]. These mulches also resulted in a remarkable decrease in the weed seed bank.

The repeated application of herbicides with same mode of action is a crucial factor involved in the rapid evolution of resistance to herbicides. To delay or avoid the evolution of resistance to herbicides, weeds may be controlled using herbicide mixtures containing two or more sites of action in rotations and/or mixtures [102]. *P. paradoxa* populations with the Asp-2078-Gly mutation in their ACCase encoding gene, collected in wheat fields in Israel, were resistant to haloxyfop-R methyl, fluazifop-P ethyl, clodinafop-propargyl propargyl, quizalofop-P-ethyl, cycloxydim, clethodim, tralkoxydim, tepraloxydim, and pinoxaden (ACCase-inhibiting herbicides) [49]. These researchers concluded that multiple resistance was not observed in the resistant plants, and they were best controlled in broad-leaved crops using propyzamide herbicide (an inhibitor of microtubule assembly). However, the application of propyzamide is limited under drought conditions, as it may persist in the soil for a considerable time. Thus, the next crop in rotation must be chosen with care. Flufenacet and iodosulfuron herbicides may be considered feasible options for the chemical management of this weed in cereals, such as wheat. *P. paradoxa* populations with Asp-2078-Gly and Ile-1781-Val mutations collected from durum wheat fields in Italy were described [50]. These populations were resistant to ACC-inhibiting herbicides, including clodinafop-propargyl, diclofop-methyl, fenoxaprop-P-ethyl, sethoxydim, tralkoxydim, and pinoxaden. The results demonstrated that these populations were successfully controlled using isoproturon; a PSII inhibitor; and ALS inhibitors, such as iodosulfuron, chlorsulfuron, and imazamethabenz, in wheat.

Various mixtures of clodinafop-propargyl, metribuzin, pinoxaden, and sulfosulfuron herbicides were tested on *P. minor* populations collected from Pakistan with resistance to fenoxaprop-P-ethyl. The results showed that, while sulfosulfuron + clodinafop-propargyl at a 100% dose had phytotoxic effects on wheat, clodinafop-propargyl + metribuzin, pinoxaden + sulfosulfuron, and pinoxaden + metribuzin mixtures at a 100% dose did not harm the crop. However, all mixtures mentioned above successfully controlled herbicide-resistant *P. minor* at a 75% dose without any phytotoxic effects on wheat [111,112].

Isoproturon-resistant *P. minor* populations collected within wheat fields from India were successfully controlled by the application of chlorotoluron, a PSII-inhibiting herbicide [104]. Additionally, herbicides such as pendimethalin, trifluralin, metolachlor, atrazine, propachlor, and terbutryne have also been reported as applicable herbicides to control isoproturon-resistant *P. minor* [113]. The isoproturon-resistant *P. minor* populations were controlled using fenoxaprop-P-ethyl, clodinafop-propargyl, sulfosulfuron, diclofop-methyl, and tralkoxydim in wheat fields [39]. *P. minor* populations with resistance to the isoproturon herbicide from the Northwestern and Northeastern Indian plain regions were effectively controlled in wheat fields by the application of fenoxaprop-P-ethyl, pinoxaden, clodinafop-propargyl, mesosulfuron-methyl, sulfosulfuron, fluazolate, and pendimethalin [114]. Although the addition of compounds such as malathion eliminated isoproturon-resistant *P. minor* populations, the application of this pesticide in the field will damage the crop as well, and thus, it is not a feasible strategy [115].

The chemical management of *P. minor* has also been successful for ACCase-resistant *P. minor* in wheat using sulfosulfuron, pinoxaden, mesosulfuron + iodosulfuron, metribuzin, and sequential application of pendimethalin + metribuzin followed by mesosulfuron + iodosulfuron, pendimethalin + metribuzin, sulfosulfuron + metsulfuron [11,116,117,118].

The resistance of *P. minor* to sulfosulfuron, sulfosulfuron + metsulfuron, and mesosulfuron + iodosulfuron herbicides was overcome by the application of pendimethalin herbicide [62]. The successful control of *P. minor* populations with resistance to multiple ACCase and ALS inhibitors in wheat has been reported using pendimethalin + pyroxasulfone, followed by mesosulfuron + iodosulfuron [119]. Metribuzin, terbutryn, and pendimethalin herbicides also controlled *P. minor* populations with a resistance to multiple ACCase and ALS inhibitors in wheat, and populations resistant to isoproturon and clodinafop-propargyl were best managed using sulfosulfuron [59]. Pinoxaden efficiently controlled multiple-resistant *P. minor* in wheat. However, it did not provide effective broadleaf weed control. The best tank mixes for the control of these populations were metribuzin + clodinafop-propargyl, metribuzin + sulfosulfuron, and trifluralin, followed by clodinafop-propargyl or sulfosulfuron [77]. Additionally, pyroxasulfone [120], flufenacet [120,121], and flumioxazin [122] have been reported to successfully control *P. minor* populations with resistance to multiple PSII, ACCase, and ALS inhibitors. The addition of sulfosulfuron + clodinafop-propargyl, clodinafop-propargyl + metribuzin, and pinoxaden + sulfosulfuron herbicides at 50% of the recommended dose had a positive effect on the control of herbicide-resistant *P. minor* in wheat and led to yield enhancement [123].

Currently, no reports are available on the management of herbicide-resistant *P. brachystachys*, and this issue requires more research. According to the results mentioned in the present paper, the differences observed between the S and R biotypes of *P. brachystachys* can be a good starting point for developing a resistance management strategy. Variations in the response of the *P. brachystachys*-resistant biotype to environmental factors can affect the competitive capacity of R biotypes over the susceptible ones with prolonged emergence, as the rapid occupation of a biological space is crucial for capturing light and avoiding competitor shading, especially when soil resources are limited, thus affecting their success in the field. Thus, by stimulating the germination of resistant biotypes or delaying planting the main crop, resistant biotypes that have emerged rapidly can be managed by tillage or the application of herbicides from other families such as ALS and PS II inhibitors to reduce the frequency of resistant biotypes to susceptible ones. Differences in vegetative traits such as height, number of tillers, leaf area, and dry weight between resistant and susceptible biotypes of *P. brachystachys* indicate that, in the absence of competition, resistant plants have a higher growth and production potential than susceptible plants. The existence of differences between the plant growth characteristics of resistant and susceptible biotypes may be important for controlling species based on the crop growth stage with post-emergence herbicides.

## 7. Conclusions and Future Directions

The evolution of herbicide resistance in weeds, such as *Phalaris* spp., poses a serious threat to sustainable agriculture. In general, herbicide families common in wheat to which the studied biotypes are susceptible may still be utilized. Furthermore, the cultivation of imazamox or glufosinate-resistant varieties of wheat [122] may be an option, as there are no reports of resistances to these herbicides in *Phalaris* spp. The registration and testing of new herbicides are also recommended. Although herbicides are indispensable weapons in the battle against weed infestations, overreliance on a single chemical option may lead to more complicated cases of herbicide resistance. Investigations into the fitness costs imposed by herbicide resistance are suggested, as the differences observed between the herbicide-susceptible and -resistant biotypes may be exploited to devise weed management strategies. Studies related to the fitness cost of herbicide resistance in *Phalaris* spp. are very limited; thus, the authors encourage weed science researchers to further explore this issue and expand integrated weed management possibilities. Crop rotation and consequently implementation of diverse weed management methods would be the most suitable approach to control herbicide-resistant weeds. Additionally, the rotation of the herbicides, as well as an application of herbicides with different modes of action (from different chemical families) are among the possible ways to control resistant *Phalaris* species in the short term. Hence, an integrated management approach is needed to fight against the evolution of herbicide resistance in *Phalaris* species. Moreover, emphasis on mapping herbicide-resistant weeds for site-specific weed management using novel technologies such as robots and drones is also recommended [124].

## Figures and Tables

**Table 1:** Summary of the current worldwide occurrence of *Phalaris* spp. with resistance mechanisms to different herbicide groups.

*Phalaris* Species	Country	First Report	Type of Resistance					Mode of Action	Resistance Mechanism	References
			ALS ^1^	PSII ^2^	APP ^3^	CHD ^4^	PPZ ^5^			
*P. minor*	Mexico	1996	-	-	R	R	R	ACCase inhibitors	Ile-1781-Leu Asp-2078-Gly Ile-2041-Asn Trp-2027-Cys	[35]
	South Africa	1999	R	-	R	-	-	Multiple Resistance: ACCase inhibitors ALS inhibitors	NE	[36]
	India	1991	-	R	-	-	-	PSII inhibitor (Ureas and amides)	Metabolism	[8,37,38,39]
		1994	-	-	R	R	r	ACCase inhibitors	Trp-2027-Cys Ile-2041-Asn	[40,41]
		2006	r	r	R	R	-	Multiple Resistance: ACCase inhibitors, ALS inhibitors, PSII inhibitor	NE	[42]
		2013	R	-	-	-	-	ALS inhibitors	NE	[43]
	United States	2001	-	-	R	R	-	ACCase inhibitors	NE	-
	Israel	1993	-	-	R	r	-	ACCase inhibitors	NE	[44]
	Australia	2012	-	-	R	-	-	ACCase inhibitors	NE	-
	Iran	2004	-	-	R	R	S/R	ACCase inhibitors	Trp-2027-Cys Asp-2078-Gly Ile-1781-Leu	[45]
	Pakistan	2015	-	-	R	-	-	ACCase inhibitors	NE	[46]
*P. paradoxa*	Australia	1997	-	-	R	R	-	ACCase inhibitors	NE	-
		2012	R	-	R	-	-	Multiple Resistance: ACCase inhibitors ALS inhibitors	NE	-
	Iran	2007	-	-	R	R	R	ACCase inhibitors	NE	-
	Israel	1979	-	R	-	-	-	Photosystem II inhibitors (atrazine)	Ser-264-Gly	[47,48]
		2004	-	-	R	R	r	ACCase inhibitors	Asp-2078-Gly Ile-2041-Asn	[49]
	Italy	1998	-	-	R	R	R	ACCase inhibitors	Ile-1781-Val Asp-2078-Gly	[50]
	Mexico	1996	-	-	R	R	R	ACCase inhibitors	Gly-2096-Ser	[51]
	Syria	2015	-	-	R	-	-	ACCase inhibitors	NE	-
*P. brachystachys*	Italy	2001	-	-	R	R	R	ACCase inhibitors	NE	-
	Turkey	2008	R	-	R	-	-	Multiple Resistance: ACCase inhibitors ALS inhibitors	NE	-
	Iran	2014	-	-	R	R	r	ACCase inhibitors	Ile-1781-Thr Metabolism	[52]
	Syria	2015	-	-	R	-	-	ACCase inhibitors	NE	-

^1^ Acetolactate synthase, ^2^ photosystem II, ^3^ APP: aryloxyphenoxypropionate, ^4^ CHD: cyclohexanedione, ^5^ PPZ: phenylpyrazoline, R: resistant, S: susceptible, r: moderately resistant, and NE: not examined.

## Data Availability

Data is contained within the article.
